# William Harvey and the discovery of the circulation of the blood

**DOI:** 10.1186/2040-2384-1-3

**Published:** 2009-09-21

**Authors:** Domenico Ribatti

**Affiliations:** 1 Department of Human Anatomy and Histology, University of Bari Medical School, Bari, Italy

## Abstract

This Commentary emphasizes the fundamental contribution of William Harvey to the discovery of the circulation of the blood and his scientific and experimental approach to this matter.

## Commentary

**Figure 1:**
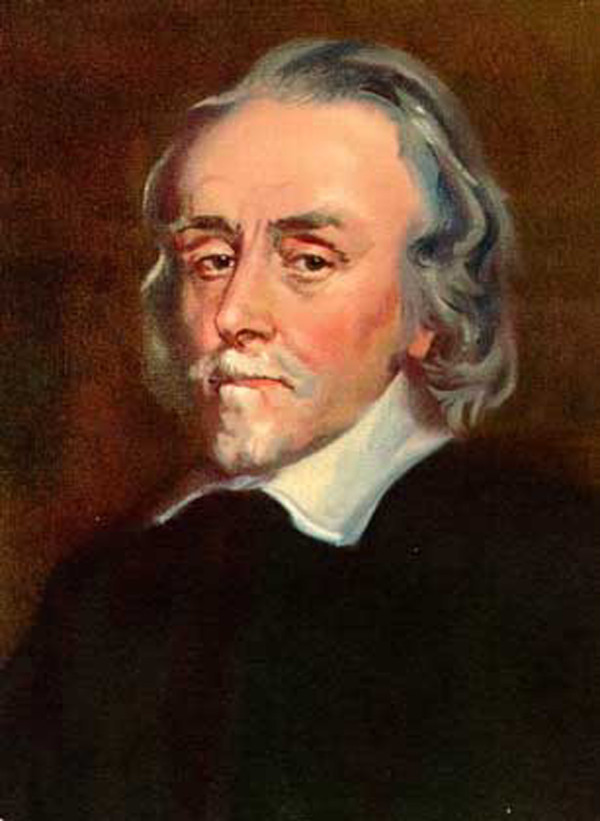
**A portrait of William Harvey**.

Harvey was born at Folkestone, Kent, England, April 1, 1578. He received the degree of Medical Doctor from the University of Padua, Italy in 1602. After his return to England he became Fellow of the College of Physicians, physician to St. Bartholomew's Hospital, and Lumleian lecturer at the College of Physicians. In 1618, Harvey was appointed physician extraordinary to James I, and he remained in close professional relations to the royal family (Figure 1). He died on June 3, 1657, at age 79. His last contribution was a book on the growth and development of the young animals entitled "De Generatione Animalium", published in 1651.

Harvey focused much of his research on the mechanics of blood flow in the human body. Most physicians of the time felt that the lungs were responsible for moving the blood around throughout the body. Harvey's famous "Exercitatio Anatomica de Motu Cordis et Sanguinis in Animalibus", commonly referred to as "de Motu Cordis" was published in Latin at Frankfurt in 1628, when Harvey was 50 years old. The first English translation did not appear until two decades later.

Harvey, observing the notion of the heart in living animals, was able to see that systole was the active phase of the heart's movement, pumping out the blood by its muscular contraction. Having perceived that the quantity of blood issuing from the heart in any given time was too much to be absorbed by the tissues, he was able to show that the valves in the veins permit the blood to flow only in the direction of the heart and to prove that the blood circulated around the body and returned to the heart. Fabricius, his teacher in Padua, had discovered the valves in the veins.

In Chapter 8 of "De Motu cordis", Harvey wrote how he hypothesized that the blood circulates: "In truth, when, from a variety of investigators through dissection of the living in order to experiment and through the opening of arteries, from the symmetry and magnitude of the ventricles of the heart and of the vessels entering and leaving (since Nature, who does nothing in vain, would not have needkessly given these vessels such relatively large size), from the skilfull and careful craftsmanship of the valves and fibres and the rest of the fabric of the heart, and from many other things, I had very often and seriously though about, and had long turned over in my mind, how great an amount there was, that is to say how great the amount of transmitted blood would be [and] in how short a time that transmission would be effected...I began privately to think that it might rather have a certain movement, as it were, in a circle..."

In Chapter 13, Harvey summarized the substance of his findings: "It has been shown by reason and experiment that blood by the beat of the ventricles flows through the lungs and heart and is pumped to the whole body. There it passes through pores in the flesh into the veins through which it returns from the periphery everywhere to the centre, from the smaller veins into the larger ones, finally coming to the vena cava and right atrium. This occurs in such an amount, with such an outflow through the arteries and such a reflux through the veins, that it cannot be supplied by the food consumed. It is also much more than is needed for nutrition. It must therefore be concluded that the blood in the animal body moves around in a circle continuously and that the action or function of the heart is to accomplish this by pumping. This is only reason for the motion and beat of the heart."

Harvey's predecessors and contemporaries believed the blood to be continually formed anew from the digested food, to be dissipated and used up in the tissues, and considered that the primary function of the heart was the production of heat. Blood was constantly being consumed in the periphery and replenished by ingested nutrients, and this was all carried out by the right ventricle and great veins. Harvey studied the hearts not only of various fishes, amphibian, reptiles, birds, and mammals, but also those of various other animal species. But most important, he not only compared these, he manipulated them in living as well as dead animals. He isolated parts of the heart; he ligated and divided arteries; he exerted pressure on veins on either side of the valves. His observations of dissected hearts showed that the valves in the heart allowed blood to flow in only one direction. Harvey measured the volume of the left ventricle and calculated that the amount of blood that passes through the heart of a man in an half hour and established that it was greater than the amount contained in the whole body. Direct observation of the heartbeat of living animals showed that the ventricles contracted together, dispelling Galen's theory that blood was forced from one ventricle to the other. Dissection of the septum of the heart showed that it contained no gaps or perforations. When Harvey removed the beating heart from a living animal, it continued to beat, thus acting as a pump, not a sucking organ. Harvey also used mathematical data to prove that the blood was not being consumed. Finally, Harvey postulated the existence of small capillary anastomoses between arteries and veins, but these were not discovered until 1661 by Marcello Malpighi.

## Competing interests

The author declares that he has no competing interests.

